# Fractalkine (CX3CL1), GM-CSF and VEGF-a levels are reduced by statins in adult patients

**DOI:** 10.1186/2001-1326-3-14

**Published:** 2014-06-14

**Authors:** Thomas R Cimato, Beth A Palka

**Affiliations:** 1 Department of Medicine/Division of Cardiovascular Medicine, School of Medicine and Biomedical Sciences, Clinical and Translational Research Center, State University of New York at Buffalo, Buffalo, NY 14203, USA

## Abstract

**Background:**

Fractalkine (CX3CL1) promotes migration and adhesion of lymphocytes and monocytes to inflamed tissues. Prior studies show a role for CX3CL1 in atherosclerosis. The relationship between inflammatory cytokines, cholesterol, and CX3CL1 levels in human subjects without known coronary artery disease is not well characterized. The goal of our study was to assess baseline CX3CL1 levels, and after modulation of cholesterol levels by statins to determine if CX3CL1 is linked to cholesterol levels or inflammatory stimuli.

**Methods:**

We performed a blinded, randomized hypothesis generating study in human subjects without known coronary artery disease treated sequentially with three statins of differing potencies. Fractalkine (CX3CL1), GM-CSF, VEGF-A, other chemokines, and lipid levels were measured. Mechanistic studies of CX3CL1 induction by LDL cholesterol and TNFα in cultured human endothelial cells were performed using real-time PCR.

**Results:**

Therapy with statins reduced total and LDL cholesterol levels as expected. CX3CL1 levels were significantly reduced from no statin control levels (89.9 ± 18.5 pg/mL) after treatment with atorvastatin (60.0 ± 7.8 pg/mL), pravastatin (54.2 ± 7.0 pg/mL) and rosuvastatin (65.6 ± 7.3 pg/mL) (*χ*^2^(2) = 17.4, p ≤ 0.001). Cholesterol is not a known regulator of CX3CL1. We found GM-CSF (r^2^ = 0.524; p < 0.005) and VEGF-A (r^2^ = 0.4; p < 0.005) levels were highly and positively correlated with CX3CL1. Total (r^2^ = 0.086) and LDL cholesterol (r^2^ = 0.059) levels weakly correlated with CX3CL1 levels. Finally, we tested whether LDL cholesterol could induce CX3CL1, GM-CSF, and VEGF-A in human endothelial cells, versus TNFα. LDL cholesterol alone resulted in small, non-significant increases in CX3CL1 and GM-CSF, while TNFα resulted in > 10-fold induction.

**Conclusions:**

Our findings suggest that while statins suppress CX3CL1 levels, inflammatory cytokines may be the major regulator of CX3CL1 levels rather than cholesterol itself. Additional studies in a larger patient population are needed to confirm these findings, determine if CX3CL1 levels reflect inflammation levels, and potentially add to standard risk factors in prediction of atherosclerotic disease events.

## Background

Chemokines are small peptides that form a chemical gradient which guides migration of inflammatory cells to sites of disease. Fractalkine (CX3CL1) is a structurally distinct chemokine. It is a membrane bound glycoprotein with a chemokine domain atop a mucin-like stalk and unlike other CX3C family chemokines, signals through a single Gαi-linked receptor: CX3CR1 [1]. Membrane bound fractalkine is induced most abundantly in endothelial cells by several inflammatory cytokines, promoting integrin independent adhesion of CD16+ CX3CR1+ monocytes [2] and CD8+ CX3CR1+ cytotoxic T lymphocytes [3]. CX3CL1 expression on inflamed endothelium attracts NK cells and cytotoxic T cells, resulting in lysis of neighboring endothelial cells [4]. Additional sources of CX3CL1 include monocytes, macrophages, fibroblasts and dendritic cells in synovial tissue, indicating a role for CX3CL1 in rheumatologic diseases as well [5]. These findings link CX3CL1 expression directly to recruitment of inflammatory cell types. CX3CL1 is also released from the cell membrane in an active soluble form by proteolysis via ADAM10 and 17. Release of soluble CX3CL1 creates a chemokine gradient guiding chemotaxis of inflammatory cells to sites of injury [5].

Several lines of evidence indicate a role for CX3CL1 in the pathogenesis of atherosclerosis. Both CX3CL1^-/-^ and CX3CR1^-/-^ knockout mice crossed into the apoE^-/-^ model of atherosclerosis showed a significant reduction in macrophage recruitment to the vessel wall and decreased atherosclerotic lesion formation compared to normal animals [6,7]. In humans, CX3CR1 polymorphisms in the coding region of the gene are a genetic risk factor for early onset coronary artery disease, strongly supporting a mechanistic role for CX3CL1 in the pathogenesis of atherosclerosis [8,9]. Both CX3CL1 levels, and CD3+CD8+ CX3CR1+ T cell levels increase in patients with chronic coronary artery disease [10] and acute coronary syndromes with plaque rupture [11], strongly supporting a link between CX3CL1 and atherosclerosis in humans.

A clear link between CX3CL1 levels, polymorphisms, and coronary artery disease in humans is well defined. While hypercholesterolemia is a known risk factor for coronary artery disease events, most subjects with acute coronary syndromes have mildly elevated cholesterol levels [12]. However, the levels of CX3CL1 in asymptomatic subjects without known coronary disease have not been studied. Prior studies indicate that CX3CL1 levels are significantly increased in both chronic coronary artery disease and acute coronary syndromes [10,11]. The effect of cholesterol lowering therapy on CX3CL1 levels in subjects with chronic coronary artery disease is less clear as the high potency statin, atorvastatin 80 mg daily reduced CX3CL1 levels, while a lower potency statin, simvastatin 20 mg daily did not decrease CX3CL1 [10]. Given the clear role of hypercholesterolemia as a risk factor for atherosclerotic diseases, and the functional role of CX3CL1 in atherosclerosis and plaque rupture, we aimed to determine if CX3CL1 levels varied with cholesterol levels in human subjects without known atherosclerotic disease. In this study we determined baseline CX3CL1 levels in human subjects without known coronary disease and after modulation of cholesterol levels using different statins to determine if CX3CL1 levels are linked to cholesterol levels or other inflammatory stimuli.

## Methods

### Patient consent for participation

Our research protocol was reviewed and approved by the University at Buffalo Intramural Review Board for Health Sciences research (Approval Number: MED5980509B). Informed consent to undergo the study protocol was obtained in writing from each participant according to the principles expressed in the Declaration of Helsinki. Our study was organized as an observational trial as no direct health outcomes were to be measured.

### Characteristics of study subjects

The study population consisted of 12 adult subjects (7 males and 5 females) with no active medical problems. Study subjects were screened for the absence of chronic health disorders including hypercholesterolemia with additional cardiovascular risk factors, cancer, diabetes, chronic liver or kidney disease [13]. The baseline characteristics of the study population were reported previously [13] and are shown in Table 1. The age of the cohort was 43.4 ± 12.5 years, had a body mass index of 24.9 ± 7.2, and were at low risk for atherosclerotic disease events with a Framingham Risk Score of 1.7 ± 0.5. Prior to statin treatment the mean total cholesterol level was 210.5 ± 27.6 mg/dL, LDL cholesterol 136.2 ± 22.9 mg/dL, HDL cholesterol 53.5 ± 12.9 mg/dL. The cohort had a low index of inflammation as the C-reactive protein level was 1.1 ± 1.3 mg/L. Two of the study subjects had treated hypertension. None of our study subjects had disease states known to increase CX3CL1 levels including common variable immunodeficiency [14], granulomatosis [15], congestive heart failure [16], and did not smoke [17].

**Table 1:** Clinical data

		**Mean values ± SD**			**p-value (statin vs. baseline)**		
	**Baseline**	**Atorvastatin**	**Pravastatin**	**Rosuvastatin**	**Atorvastatin**	**Pravastatin**	**Rosuvastatin**
**Age**	43.4 ± 12.5						
**BMI**	24.9 ± 7.2						
**Framingham Risk Score**	1.7 ± 0.5						
**Total Cholesterol (mg/dL)**	210.5 ± 27.6	138.5 ± 28.9	160.6 ± 28.9	154.2 ± 21.0	<0.0001	<0.0001	<0.0001
**LDL Cholesterol (mg/dL)**	136.2 ± 22.9	68.2 ± 12.1	88.7 ± 24	83.3 ± 12.7	<0.0001	<0.0002	<0.0001
**HDL Cholesterol (mg/dL)**	53.5 ± 12.9	54.1 ± 18.6	54.5 ± 13.1	55.3 ± 15.3	NS	NS	NS
**C-Reactive Protein (mg/L)**	1.1 ± 1.3	1.05 ± 1.1	0.95 ± 0.8	1.3 ± 1.5	NS	NS	NS

### Study protocol

**Figure 1:**
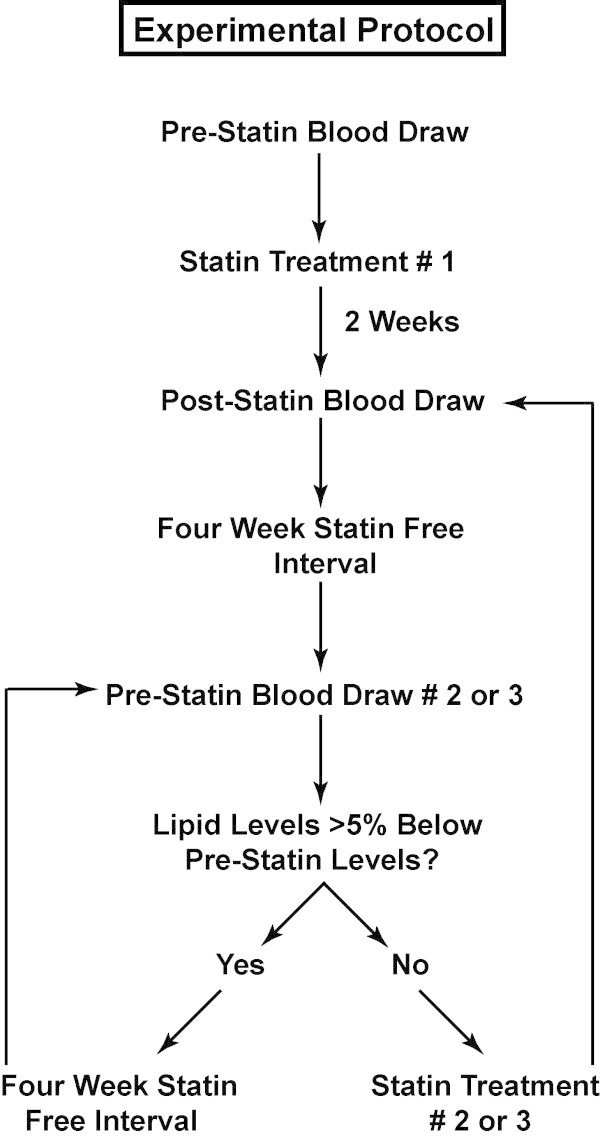
Flow chart of experimental design.

Study subjects were randomized to drug regimen groups using a block randomization design. The investigators were blinded to which treatment subjects were receiving. The workflow of the experimental protocol is summarized in Figure 1. Study subjects underwent a baseline blood draw in which a complete blood cell count, lipid panel (total, HDL, and LDL cholesterol, and triglycerides) and C-reactive protein were determined by the Kaleida Health pathology laboratory. The plasma fraction was also retained from each blood draw and frozen at -80°C for chemokine and cytokine assays. Subjects were then treated for two weeks with one of three different HMG-CoA reductase inhibitors (pravastatin 80 mg daily, atorvastatin 80 mg daily, or rosuvastatin 10 mg daily). At the end of the two-week statin treatment, venous blood was sampled again to obtain the lipid panel, C-reactive protein level, and blood samples for cytokine analysis. Subjects were subsequently given a four-week statin free period. At the end of the four week statin-free period, venous blood was sampled again to determine if serum lipids returned to within 5% of their pre-statin levels. In subjects where cholesterol levels did not recover to within 5% of the baseline lipid levels, an additional four-week statin free period was provided before resuming statin therapy to avoid effects of overlap between drugs. Three study subjects required extension of the statin free period for an additional four weeks for serum lipid levels to return to within 5% of their baseline lipid levels. Following this period, the next HMG-CoA reductase inhibitor in the randomization scheme was given for two weeks. The same protocol was repeated for statin drugs two and three until study completion. All twelve subjects completed treatment with the three statin medications.

### Cytokine and chemokine assays

Plasma levels of fractalkine (CX3CL1), GM-CSF, FLT3 ligand, GRO alpha, Interleukin-3, Interleukin-6, Interleukin-8, macrophage inflammatory protein-1 (MIP-1/CCL3), monocyte chemotactic protein-1 (MCP-1), stromal derived growth factor-1 (SDF-1), and VEGF-A were measured with a Luminex Human Cytokine assay (Millipore) per the manufacturer’s instructions by the Roswell Park Cancer Institute Laboratory of Flow Cytometry. For Luminex based assays, known concentrations of CX3CL1, GM-CSF, and VEGF-A were measured in triplicate to generate standard curves using a non-linear five-parameter curve fit using the nCal package in R [18]. The reliable lower assay limits for each were: CX3CL1-39.5 pg/mL, GM-CSF 3.02 pg/mL, VEGF-A 36.6 pg/mL. The inter-assay %CV for CX3CL1 was 5.13, and the intra-assay %CV for CX3CL1 was 5.85. Plasma cytokine levels from human subjects before and after statin treatment were measured in singulate. Interleukin-17 levels were measured with an ELISA (R&D Systems).

### Real time PCR analysis

Human umbilical vein endothelial cells (Lonza, passages 3–5) were grown to 80% confluence in EGM-2 medium (Lonza) on type I collagen coated culture dishes. Where indicated, cells were incubated for 18 hours in human LDL cholesterol (100 μg/mL; Biomedical Technologies, Stoughton, MA, USA) and/or atorvastatin (100 nM; Sigma Scientific). TNFα (1 μg/mL; R&D Systems) was then added for an additional 18 hours after LDL cholesterol or atorvastatin treatment where indicated. Cells were then washed in warm PBS and lysed in QIAzol solution (Qiagen), and RNA was extracted following the manufacturer’s protocol. RNA quality was assessed by OD260/280 using a Nanodrop spectrophotometer. Total RNA was reverse transcribed using SuperScript III Reverse Transcriptase with oligo-dT primers (Invitrogen). Real-time PCR analysis was performed using SYBR green and Taq DNA polymerase (Qiagen) on a Bio-Rad CFX96 Connect Real-Time PCR Detection System. PCR primers for CX3CL1, GM-CSF, ICAM, VCAM, CX3CR1, and VEGF-A were obtained from Qiagen. RT-PCR data was normalized to GAPDH expression to identify relative changes in transcript levels.

### Statistical analysis

Regression analyses were tested for significance using Pearson’s correlation. Heat map representations of RT-PCR data were compiled using R. Significant differences in CX3CL1, GM-CSF, and VEGF-A plasma levels between statin therapies were assessed using a non-parametric Friedman’s two-way ANOVA by ranks with a Bonferroni correction for multiple comparisons. Differences in CX3CL1, ICAM, VCAM-1, and VEGF-A transcript levels in response to LDL cholesterol incubation or TNFα versus untreated cells were measured by a one-way ANOVA with a Bonferroni correction for multiple comparisons. RT-PCR data for GM-CSF was analyzed by one-way ANOVA with a Games-Howell post-hoc test due to unequal variances between treatment groups. Numerical data stated in the manuscript text represent mean ± standard error of the mean. Statistical analysis was performed using SPSS software.

## Results

### Effects of statins on CX3CL1 levels in human subjects without known heart disease

**Figure 2:**
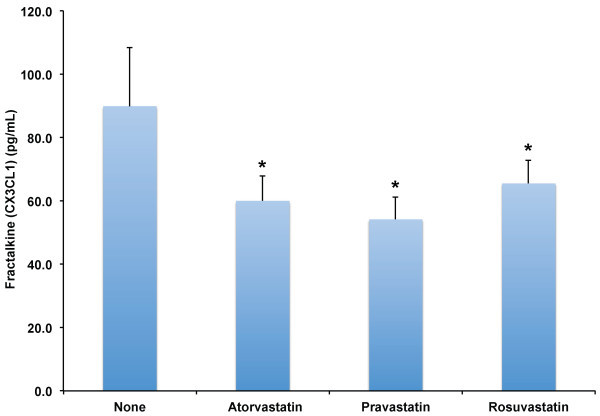
**Effect of HMG-CoA reductase inhibitors on fractalkine (CX3CL1) plasma levels in adult human subjects.** Levels of Fractalkine (CX3CL1) were determined in plasma of adult human subjects (n = 12) without known coronary artery disease after no therapy (None), Atorvastatin 80 mg daily, Pravastatin 80 mg daily, or Rosuvastatin 10 mg daily for two weeks. Results shown are the mean values and error bars indicate standard error of the mean. * indicate significant differences versus no statin therapy, p ≤ 0.001.

To understand whether CX3CL1 levels vary with cholesterol levels or other factors in humans, we assessed CX3CL1 levels in human subjects without known coronary disease and used HMG-CoA reductase inhibitors (statins) of varying potencies to modulate cholesterol levels. The baseline characteristics of the study cohort were published previously [13], and are shown in Table 1. Baseline serum lipids revealed an average total cholesterol of 210.5 ± 27.6 mg/dL (range 168 to 282 mg/dL) and an average LDL cholesterol of 136.2 ± 22.9 (range 114 to 205). The total and LDL cholesterol levels spanned the normal and hypercholesterolemic range. Therapy with statins reduced total and LDL cholesterol levels as expected, demonstrating appropriate drug effect [13]. CX3CL1 levels were significantly reduced from no statin control levels (89.9 ± 18.5 pg/mL) after treatment with atorvastatin (60.0 ± 7.8 pg/mL), pravastatin (54.2 ± 7.0 pg/mL) and rosuvastatin (65.6 ± 7.3 pg/mL) (*χ*^2^(2) = 17.4, p ≤ 0.001; see Figure 2), indicating statins downregulate CX3CL1 levels in adult human subjects.

### GM-CSF and VEGF-a levels are positively correlated with CX3CL1 levels

**Figure 3:**
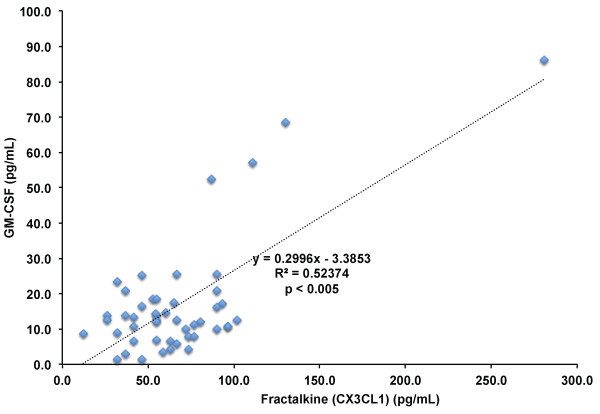
**Correlation of fractalkine (CX3CL1) levels and GM-CSF levels in blood of adult human subjects.** Fractalkine (CX3CL1) and GM-CSF plasma levels measured in all subjects (n = 12 subjects) before and after statin treatments. p ≤ 0.005 by Pearson’s correlation.

**Figure 4:**
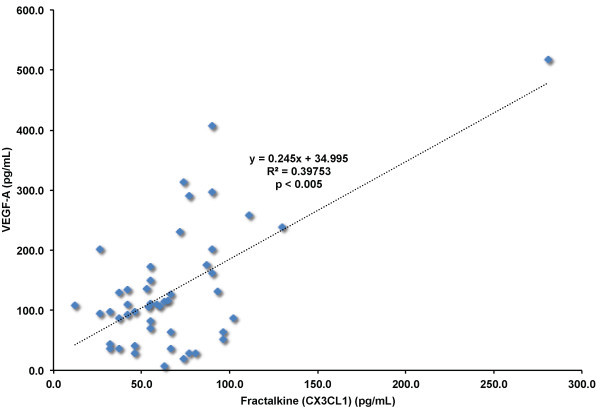
**Correlation of fractalkine (CX3CL1) levels and VEGF-A levels in blood of adult human subjects.** Fractalkine (CX3CL1) and VEGF-A plasma levels measured in all subjects (n = 12 subjects) before and after statin treatments. p ≤ 0.005 by Pearson’s correlation.

**Figure 5:**
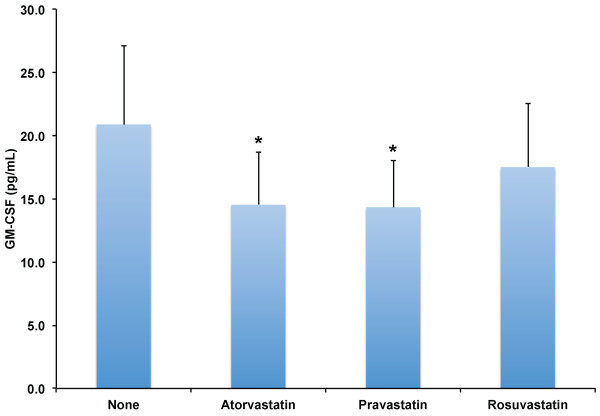
**Effect of HMG-CoA reductase inhibitors on GM-CSF plasma levels in adult human subjects.** Levels of GM-CSF were determined in plasma of adult human subjects without known coronary artery disease (n = 12) after no therapy (None), Atorvastatin 80 mg daily, Pravastatin 80 mg daily, or Rosuvastatin 10 mg daily for two weeks. Results shown are the mean values and error bars indicate standard error of the mean. *indicate significant differences versus no statin therapy, p ≤ 0.005.

**Figure 6:**
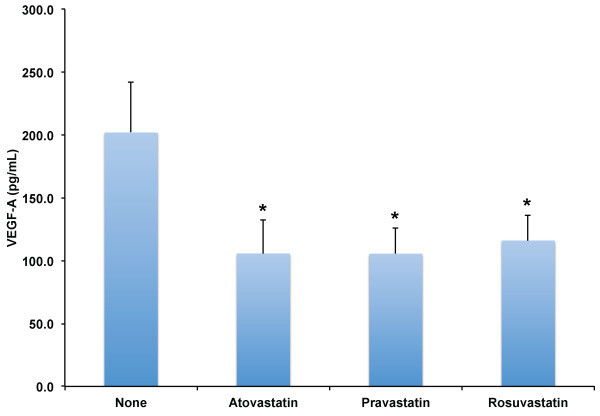
**Effect of HMG-CoA reductase inhibitors on VEGF-A plasma levels in adult human subjects.** Levels of VEGF-A were determined in plasma of adult human subjects without known coronary artery disease (n = 12) after no therapy (None), Atorvastatin 80 mg daily, Pravastatin 80 mg daily, or Rosuvastatin 10 mg daily for two weeks. Results shown are the mean values and error bars indicate standard error of the mean. *indicate significant differences versus no statin therapy, p ≤ 0.05.

We next sought to elucidate factors regulating CX3CL1 after statin therapy. CX3CL1 is induced by inflammatory cytokines including interferon-γ, interleukin-6, and TNFα [5]. Cholesterol however, is not a known regulator of CX3CL1. We surveyed the levels of inflammatory cytokines in the plasma of our cohort before and after statin therapy. We found FLT3 ligand, Interleukin-3, Interleukin-6, and Macrophage inflammatory protein-1 (MIP-1/CCL3) were undetectable in most subjects, indicating a lack of typical CX3CL1 inducers in our cohort of asymptomatic human subjects. We identified correlations between CX3CL1 plasma levels and granulocyte macrophage-colony stimulating factor (GM-CSF), VEGF-A, stromal cell-derived factor-1/CXCL12 (SDF-1), GROα/CXCL1, monocyte chemotactic protein-1/CCL2 (MCP-1), interleukin-8/CXCL8 (IL-8), neutrophil counts, and cholesterol levels. We performed regression analysis to test significant correlations with CX3CL1 levels in human blood (Table 2). We found that GM-CSF (r^2^ = 0.524; p < 0.005; Figure 3) and VEGF-A (r^2^ = 0.4; p < 0.005; Figure 4) levels were highly and positively correlated with CX3CL1. To explore why GM-CSF and VEGF-A were significantly correlated with CX3CL1 levels, we evaluated if statins decreased the concentrations of GM-CSF and VEGF-A, and if GM-CSF and VEGF-A levels correlated with total, LDL, or HDL cholesterol values. We found that all statin therapies significantly decreased both GM-CSF (Figure 5) and VEGF-A levels (Figure 6), but there was no significant correlation between GM-CSF or VEGF-A levels with total, LDL, or HDL cholesterol. These findings suggest that the effect of statins on GM-CSF and VEGF-A levels are more likely driven by suppression of inflammatory signaling rather than a reduction in cholesterol levels.

**Table 2:** Correlates with fractalkine levels

**Covariate**	**Regression coefficient**	**Positive or negative**	**P-value**
**GM-CSF**	**0.524**	**Positive**	**< 0.005**
**VEGF-A**	**0.4**	**Positive**	**< 0.005**
**IL-8/CXCL8**	**0.239**	**Positive**	**< 0.005**
**SDF-1/CXCL12**	**0.125**	**Positive**	**< 0.005**
**Neutrophils**	**0.1**	**Positive**	**< 0.005**
**LDL Cholesterol**	**0.086**	**Positive**	**< 0.025**
**GRO alpha/CXCL1**	**0.086**	**Negative**	**< 0.025**
**HDL Cholesterol**	**0.085**	**Negative**	**< 0.025**
**MCP-1/CCL2**	**0.061**	**Positive**	**< 0.05**
**Total Cholesterol**	**0.059**	**Positive**	**< 0.05**
**C-reactive protein**	**0.026**	**Positive**	**> 0.1**
**G-CSF**	**0.007**	**Positive**	**> 0.1**
**VEGF-C**	**0.004**	**Positive**	**> 0.1**
**CD34+ CD45 dim**	**0.003**	**Positive**	**> 0.1**
**Interleukin-17**	**0.00008**	**Positive**	**> 0.1**

**Figure 7:**
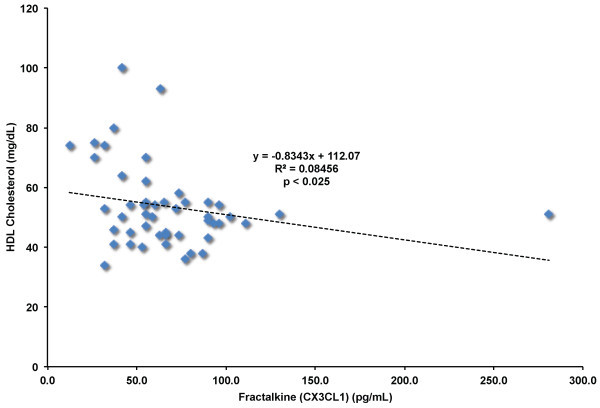
**Correlation of fractalkine (CX3CL1) levels and HDL cholesterol levels in blood of adult human subjects.** Fractalkine (CX3CL1) and HDL cholesterol levels measured in all subjects (n = 12) before and after statin treatments. p ≤ 0.025 by Pearson’s correlation.

SDF-1/CXCL12, LDL cholesterol, MCP-1/CCL2, total cholesterol, and IL-8/CXCL8 levels all showed statistically significant associations with CX3CL1 levels. GROα/CXCL1, circulating neutrophil levels, and HDL cholesterol levels (Figure 7) were negatively correlated with CX3CL1 levels. We did not find significant correlation between CX3CL1 levels and C-reactive protein, IL-17, CD34+ HSPC levels, VEGF-C or G-CSF. Interestingly, the shared biological function of GM-CSF, VEGF-A, SDF-1, GROα, and IL-8 lies in regulation of chemotaxis and response to inflammation. These findings suggest that CX3CL1, along with several other factors involved in response to inflammation and chemotaxis of inflammatory cell types are modulated by statin therapy.

### LDL cholesterol itself weakly induces CX3CL1, GM-CSF, and VEGF-a while statins suppress their expression in human endothelial cells

**Figure 8:**
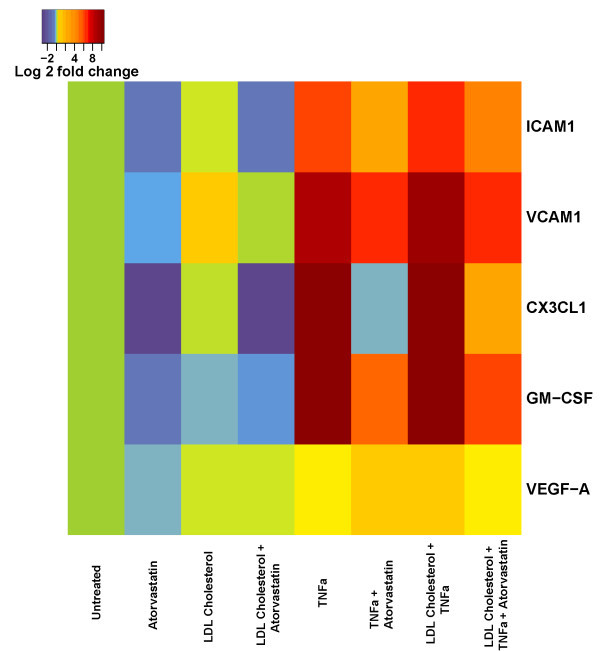
**Heat map plot of real-time PCR data of GM-CSF, fractalkine (CX3CL1), VCAM1, ICAM1, and VEGF-A in human endothelial cells stimulated with LDL cholesterol and TNFα.** Expression of each transcript was determined and normalized to GAPDH expression. Levels of each transcript in untreated cells were arbitrarily set to zero. Data shown represent log 2-fold change in expression relative to untreated cells. Results are an average of three experimental replicates. Significant differences are noted in the Results section of the manuscript.

To determine whether CX3CL1, GM-CSF and VEGF-A levels are modulated by LDL cholesterol itself or by inflammatory cytokines, and how statins may affect CX3CL1, GM-CSF and VEGF-A expression, we incubated human umbilical vein endothelial cells with loaded with LDL cholesterol with or without atorvastatin (100 nM) for 18 hours. Then TNFα was added for an additional 18 hours. Real-time PCR analysis was performed to measure changes in the levels of CX3CL1, GM-CSF, and VEGF-A. ICAM and VCAM-1 are induced by TNFα treatment of endothelium via an NFκB dependent mechanism [19], and were positive experimental controls. Experimental results are summarized in Figure 8 showing log 2 fold changes in gene expression relative to untreated endothelial cells. We found that LDL cholesterol alone resulted in small increases in CX3CL1, ICAM1, and VCAM1, but were not significant due to a high degree of variability between experimental replicates. GM-CSF expression was modestly decreased by incubation with LDL cholesterol. LDL cholesterol incubation alone caused modest but significant increase in VEGF-A (0.1 fold increase versus untreated cells; p ≤ 0.05). TNFα treatment resulted in > 10-fold increases in transcripts of CX3CL1 (99.0 ± 0.11 fold vs. untreated; p < 0.0005), and GM-CSF (106.1 ± 1.3 fold vs. untreated; p < 0.0005). Positive controls for TNFα treatment, ICAM1 (39.7 ± 0.06 vs. untreated; p < 0.0005) and VCAM1 (82.8 ± 0.53 vs. untreated; p < 0.0005), also showed > 10-fold induction as expected. In contrast, TNFα treatment resulted in minor increases in VEGF-A (0.29 ± 0.01 vs. untreated; p < 0.006) transcript levels. Incubation with TNFα and LDL cholesterol together resulted in modest, but not significant, augmentation of expression of CX3CL1, GM-CSF, VCAM1, ICAM1, and VEGF-A compared with TNFα treatment alone. Finally, addition of atorvastatin (100 nM) significantly reduced induction of ICAM1, VCAM1, CX3CL1 and GM-CSF by both LDL cholesterol and TNFα. In conclusion, LDL cholesterol alone did not substantially augment CX3CL1 or GM-CSF transcript levels in human endothelial cells in vitro. TNFα coordinately increased transcript levels of CX3CL1 and GM-CSF, and the addition of LDL cholesterol modestly increased both transcripts, while atorvastatin significantly reduced CX3CL1 and GM-CSF expression. The findings support a model where inflammatory cytokines intersect with cholesterol levels to augment expression of a module of inflammatory response factors including CX3CL1 and GM-CSF, and statins inhibit their induction.

## Discussion

Our study establishes the following findings: 1) In a cohort of human subjects without known coronary artery disease and cholesterol levels that span the normal and hypercholesterolemic range, CX3CL1, GM-CSF, and VEGF-A levels are significantly reduced by multiple statin therapies (atorvastatin, pravastatin and rosuvastatin). 2) CX3CL1 levels are weakly, positively correlated with statin effects on LDL cholesterol, and negatively correlate with HDL cholesterol levels. 3) CX3CL1 levels are significantly correlated with GM-CSF, VEGF-A, IL-8/CXCL8, and SDF-1/CXCL12, suggesting that this combination of chemokine factors is coordinately regulated. 4) In vitro, LDL cholesterol alone did not significantly induce CX3CL1 or GM-CSF, but had minor effects on VEGF-A expression; CX3CL1 and GM-CSF were coordinately induced by TNFα in endothelial cells, likely due to a shared mechanism regulating their expression.

Prior studies of CX3CL1 in human subjects focused on cohorts with chronic coronary artery disease, or acute coronary syndromes. Our study uniquely shows positive correlation between CX3CL1 plasma protein levels and LDL cholesterol and negative correlation with HDL cholesterol. The implied connection between LDL cholesterol levels and CX3CL1 plasma levels is important in that CX3CL1 is a functional biomarker of inflamed endothelium, which augments chemotaxis of inflammatory cells to sites of injury. Evidence from CX3CL1^-/-^[6], CX3CR1^-/-^[7], and CX3CR1 pharmacologic inhibition studies in mice [20] with a background of hypercholesterolemia mechanistically tied CX3CL1 to atherosclerosis. Taken together, these results, the mechanistic findings in animal models of atherosclerosis, and our correlative findings between LDL cholesterol and CX3CL1 levels in humans, suggest that CX3CL1 may represent a biomarker of the inflammatory component of atherosclerosis and potentially define subjects at increased risk for atherosclerotic disease events. Future studies will aim to test the relationship between CX3CL1 levels and atherosclerotic disease events and test the hypothesis that targeting CX3CL1 levels may reduce the number of atherosclerosis disease events.

Prior studies of CX3CL1 in human subjects showed substantial increases in CX3CL1 serum protein levels in the setting of chronic coronary artery disease and unstable angina. Damas and co-workers did not report a relationship between CX3CL1 protein levels and cholesterol but did show a significant positive correlation between CX3CR1 mRNA levels in peripheral blood mononuclear cells and LDL cholesterol levels [10]. In contrast to our findings, Damas and co-workers found that atorvastatin but not simvastatin decreased plasma levels of CX3CL1, while we found effectiveness of all statins tested in reducing CX3CL1 plasma levels. This may be explained by a relative lack of inflammation in our study cohort in comparison to the study by Damas et al. which focused on subjects with chronic coronary artery disease. Franco and co-workers identified significant positive correlations between CX3CL1 plasma levels and IL-6, apolipoprotein-B, LDL cholesterol, and insulin in a community based cohort of 3306 middle aged women, lending additional support to our findings in a much smaller patient population [21].

Interestingly, we found CX3CL1 levels to have a high positive correlation with GM-CSF and VEGF-A. We were unable identify any clear connection between GM-CSF and CX3CL1 in the literature. Our in vitro experiments indicate that both CX3CL1 and GM-CSF may be regulated coordinately by inflammatory cytokines such as TNFα, as both transcripts were equivalently induced. We observed that CX3CL1 protein levels were not correlated 1:1 with GM-CSF in human plasma. One possible reason for the lack of unity between the two proteins is GM-CSF mRNA is post-transcriptionally regulated and either rapidly degraded or stabilized depending on the stimulating factors [22], potentially decreasing translation of GM-CSF mRNA to protein. Interestingly, GM-CSF signaling significantly increases IL-1β secretion [23] and IL-1β is implicated in atherosclerosis in animal studies [24]. Neutralization of IL-1β is the subject of a current clinical trial (NCT01327846) to prevent recurrent cardiovascular events in patients with a prior myocardial infarction [25]. The example of GM-CSF mediated induction of IL-1β, a known mediator of atherosclerosis, provides an illustration of how CX3CL1 and GM-CSF may have multiple downstream effects to augment atherosclerosis.

We also noted a significant positive correlation between CX3CL1 and VEGF-A levels in adult human subjects (Figure 3; r^2^ = 0.4; p ≤ 0.005). In addition to recruiting inflammatory cells to sites of inflammation, CX3CL1 stimulates angiogenesis through expression of VEGF-A in endothelial cells, promoting increased proliferation and angiogenic activity in vivo [26]. Our RT-PCR experiments did not reveal that VEGF-A was significantly induced by stimulation with the inflammatory cytokine TNFα, and LDL cholesterol minimally increased VEGF-A in human endothelial cells. The correlation we identified between CX3CL1 and VEGF-A levels in the blood of adult human subjects may be tied to CX3CL1 induction of VEGF-A as previously described [26]. Additionally, we found that therapy with all three statins reduced VEGF-A levels in our study cohort, which is in agreement with prior studies in subjects with hypercholesterolemia [27], chronic coronary artery disease [28], and acute myocardial infarction [29].

We noted significant positive correlations between CX3CL1 and IL-8 levels. IL-8/CXCL8 is a proinflammatory cytokine that is produced by endothelial cells, monocytes, and vascular smooth muscle cells, and plays an important role in the migration of monocytes into the subendothelial space [30]. This represents a crucial step in initiation of atherosclerosis. In the EPIC-Norfolk Perspective Population Study, elevated blood levels of IL-8 in 785 healthy men and women predicted future coronary artery disease events after adjustment for traditional risk factors, C-reactive protein, and white cell count. IL-8 is also known as neutrophil chemotactic factor, and interestingly we noted positive correlation between CX3CL1 levels and neutrophil counts in our study. Elevated neutrophil counts themselves have been implicated as predictors of ischemic heart disease events in several epidemiologic studies [31-34]. Animal model studies indicate that hypercholesterolemia increases neutrophil counts and that neutrophils infiltrate arteries in the early stages of atherosclerosis [35]. Collectively, the signals that elevate CX3CL1 levels coincide with increased IL-8 and neutrophil levels, which are mechanistically tied to atherosclerosis.

In conclusion, we found that CX3CL1, GM-CSF, and VEGF-A levels are significantly decreased by statin therapy in adult human subjects without known coronary artery disease. CX3CL1 levels correlate with several cytokine and chemokine mediators of inflammation and inflammatory cell mobilization, as well as cholesterol levels. Mechanistically, CX3CL1 and GM-CSF transcripts were not significantly induced by cholesterol alone, but by the inflammatory cytokine TNFα. Statin therapy suppressed their protein levels in the blood of adult human subjects, and transcript levels in human endothelial cells in vitro. The effect of statins on CX3CL1 and GM-CSF levels may not be completely attributed to reduction in cholesterol levels based on our findings. Our work indicates that CX3CL1 and GM-CSF may represent putative biomarkers of inflammation associated with atherosclerosis, such as C-reactive protein, but are mechanistically tied to atherosclerosis based on the well established findings in animal models of atherosclerosis. Future studies will examine the relationship between CX3CL1 levels and atherosclerotic disease events, and assess whether targeting CX3CL1 levels would represent an effective means to reduce atherosclerosis disease events.

## Conclusions

Statin therapy significantly reduces the levels of CX3CL1 in the blood stream of human subjects without known coronary artery disease. CX3CL1 levels are weakly, but positively correlated with total and LDL cholesterol levels, and negatively correlated with HDL cholesterol levels. Remarkably GM-CSF and VEGF-A levels were highly correlated to CX3CL1 levels. In cultured human endothelial cells, LDL cholesterol alone did not result in significant induction of CX3CL1 mRNA, but TNFa coordinately induced CX3CL1 and GM-CSF mRNAs. Our findings suggest that while statins may suppress CX3CL1 levels, inflammatory cytokines may be the major regulator of CX3CL1 levels rather than cholesterol itself. Additional studies are needed to confirm these findings, and determine if CX3CL1 levels reflect inflammation levels.

## Abbreviations

CX3CL1: Fractalkine; CX3CR1: Fractalkine receptor; Statins: HMG-CoA reductase inhibitors; G-CSF: Granulocyte colony stimulating factor; GM-CSF: Granulocyte monocyte colony stimulating factor; TNFα: Tumor necrosis factor-alpha; VEGF-A: Vascular endothelial growth factor-A.

## Competing interests

The authors declare no competing interests.

## Authors’ contributions

TRC conceived and designed the study, obtained IRB approval, recruited patients and collected blood samples, collected and analyzed data, grew endothelial cell cultures, and wrote the manuscript. BAP performed data collection and entry, performed cytokine analyses and RT-PCR, performed statistical analyses, and wrote portions, and edited the entire manuscript. Both authors read and approved the final manuscript.
